# Real-Time Measurement of Volatile Chemicals Released by Bed Bugs during Mating Activities

**DOI:** 10.1371/journal.pone.0050981

**Published:** 2012-12-05

**Authors:** Ole Kilpinen, Dezhao Liu, Anders Peter S. Adamsen

**Affiliations:** 1 Department of Agroecology, Faculty of Science and Technology, Aarhus University, Slagelse, Denmark; 2 Department of Engineering, Faculty of Science and Technology, Aarhus University, Tjele, Denmark; New Mexico State University, United States of America

## Abstract

In recent years, bed bug (Hemiptera: Cimicidae) problems have increased dramatically in many parts of the world, leading to a renewed interest in their chemical ecology. Most studies of bed bug semiochemicals have been based on the collection of volatiles over a period of time followed by chemical analysis. Here we present for the first time, a combination of proton transfer reaction mass spectrometry and video analysis for real-time measurement of semiochemicals emitted by isolated groups of bed bugs during specific behavioural activities. The most distinct peaks in the proton transfer reaction mass spectrometry recordings were always observed close to the termination of mating attempts, corresponding to the defensive emissions that bed bugs have been suspected to exploit for prevention of unwanted copulations. The main components of these emissions were (*E*)-2-hexenal and (*E*)-2-octenal recorded in ratios between 1∶3 and 3∶1. In the current study, the quantity varied over 1000 fold for both of the compounds with up to 40 µg total release in a single emission. Males also emit defensive compounds due to homosexual copulation attempts by other males, and no significant differences were observed in the ratio or the amount of the two components released from males or females. In summary, this study has demonstrated that combining proton-transfer-reaction mass spectrometry with video analysis can provide detailed information about semiochemicals emitted during specific behavioural activities.

## Introduction

Bed bugs, *Cimex lectularius* L., have lived in human dwellings for thousands of years; even the ancient Egyptians knew about the great annoyance that these blood-sucking ectoparasites may cause [1]. Bed bugs are nocturnal parasites spending most of the time hidden in narrow harbourages close to the resting places of the host and only coming out to feed during the night, at intervals of several days. Most of the development from egg to adult takes place in the harbourages and often bed bugs are found in clusters due to their aggregation behaviour [2].

The study of the chemical ecology of bed bugs was initiated several decades ago but the recent resurgence in bed bug problems in many parts of the world has led to a renewed interest [3]. It is well known that bed bugs produce a characteristic smell [2] and that the main components of these volatile emissions are (*E*)-2-hexenal and (*E*)-2-octenal [4], [5]. In isolated scent glands (dorsal abdominal glands in nymphs and metathoracic glands in adults) the two main components, (*E*)-2-hexenal and (*E*)-2-octenal, are present in the ratio 3∶1 in adults and 3∶7 in 4th and 5th instar nymphs, along with smaller amounts of 2-butanone and acetaldehyde [5]. Late instar nymphs produce in addition two oxygenated aldehydes: 4-oxo-(*E*)-2-hexenal and 4-oxo-(*E*)-2-octenal [6], [7]. However, collection of volatiles emitted by bed bugs under stress has shown significant differences between females and males: females emit approximately equal amounts of (*E*)-2-hexenal and (*E*)-2-octenal, whereas males emit the two components in a ratio close to 2∶1 [7]. Behavioural studies have implicated that (*E*)-2-hexenal and (*E*)-2-octenal function as alarm pheromones inducing dispersal of bed bug aggregations [8] and that they are involved in mating behaviour [7], [9]. Bed bugs have an unusual mating behaviour where the male grasps the female from the dorsal side and penetrates the ventral surface of abdomen with the intromittent organ, depositing the sperm directly into the abdominal cavity of the female [2]. Male bed bugs will mount females irrespective of their previous mating history and multiple matings can lead to a reduced life span of the female leading to a sexual conflict between males and females [10]. Females are known to emit chemicals during male copulation attempts but the exact nature of the volatiles have not been investigated and they were not found to have any significant effect on the mating duration [11]. Experiments involving females with their scent glands blocked have shown that exposure to a mixture of (*E*)-2-hexenal and (*E*)-2-octenal in a 2∶5 ratio, which according to this study should correspond to the ratio emitted by nymphs, can deter males from mating with manipulated females, whereas ratios of 1∶1 and 5∶4 (male and female specific ratios) did not have the same effect [12]. Under laboratory conditions, male bed bugs might even attempt to mount late instar nymphs [12] or other males [9]. During such unproductive and, for the receiver, potentially dangerous copulation attempts [9], emission of volatile chemicals has also been observed. Mechanical blocking of the scent glands of last instar nymphs or males leads to increased duration of mountings directed against them, as compared to intact individuals [9], [12]. Thus, indicating that the content of the scent glands (including (*E*)-2-hexenal and (*E*)-2-octenal) function as defence against mounting attempts from males.

Collection of volatiles from jars with isolated groups of males, females, or juvenile bed bugs have shown the presence of (*E*)-2-hexenal and (*E*)-2-octenal, along with a number of other compounds in smaller amounts [13]. Ten of these compounds, including (*E*)-2-hexenal and (*E*)-2-octenal, were essential components of the bed bug aggregation pheromone. It was also shown that isolated groups of males release approximately five times more (*E*)-2-hexenal and (*E*)-2-octenal than females and at least 50 times more than juveniles, and that the ratio of the two aldehydes, in this situation, was close to 1∶10 for both males and females [13].

Previous studies on identification of pheromones or other semiochemicals released by insects have mainly been based on coupled gas chromatography and mass spectrometry (GC-MS) analysis often after collecting the volatiles on polymer sorbents [14]. One limitation of these methods is that the temporal resolution of the semiochemical release is lost, because the volatiles have to be collected over a certain time period, i.e. several minutes. Proton transfer reaction mass spectrometry (PTR-MS) is a relatively new technique [15] that has been applied in areas such as atmospheric chemistry, food and flavour science, and plant studies (see [16] for a review). In the field of insect chemical ecology, PTR-MS has been applied for odour plume tracking [17], detection of herbivore induced plant volatiles [18], and characterization of stimuli for insect electrophysiology [19]. The PTR-MS technique has the advantage that most volatile compounds present in the air sample can be detected continuously with high sensitivity and a fast time response (1–10 s). However, identification of volatiles can be difficult particularly when several compounds resulting in overlapping fractionation patterns are present simultaneously [16]. Thus, the PTR-MS technique has its most important application as a quantitative tool for measuring concentration changes over time of already identified volatiles.

In the present study, PTR-MS analysis was, for the first time, combined with video analysis and GC-MS for the study of volatile chemicals released by bed bugs during specific behavioural patterns with particular focus on the defensive emissions during mating attempts. The hypothesis was that observations at high temporal resolution could reveal the composition of these pheromones in more details and thereby settle both the dispute over the ratio of the two components and whether there are significant differences between male and female emissions. Such differences could explain earlier observations that male emissions interrupt homosexual interactions [9] whereas female emissions have been reported to have no effect on heterosexual mating attempts [11].

## Materials and Methods

### Insects

Bed bugs were taken from a laboratory population originally collected in 2006 and since then kept by regularly feeding on a human volunteer (one of the authors, thus ethics approval from the Institutional Review Board was not necessary). Between feedings, the bed bugs were kept in ventilated containers at ambient room temperature and light conditions. All bed bugs used in the experiments were adults and had been fed 5–10 days earlier to ensure that they were ready to feed during the experiments.

**Table 1:** Fragmentation pattern in the PTR-MS.

Compound	Ion masses (relative abundance)
Acetone	59 (100)
Propanal	59 (100), 31 (18)
Hexanal	55 (100), 83 (74), 101 (4), 53 (2)
(*E*)-2-Hexenal	57 (100), 99 (23), 81 (21), 43 (6)
Heptanal	55 (100), 97 (57), 69 (9), 115 (4), 53 (2)
Octanal	69 (100), 41 (47), 111 (26), 55 (11), 71 (7), 129 (6), 67 (2)
(*E*)-2-Octenal	109 (100), 57 (53), 127 (33), 67 (28), 59 (4), 83 (2)
Nonanal	69 (100), 83 (33), 55 (32), 57 (24), 143 (9), 125 (7), 71 (6), 67 (4)
Decanal	83 (100), 55 (92), 69 (22), 97 (20), 157 (13), 81 (8), 139 (2), 53 (2)
Undecanal	55 (100), 43 (66), 97 (51), 83 (38), 69 (34), 171 (19), 111 (8), 81 (3), 53 (2)
Sulcatone	109 (100), 127 (25), 69 (3), 67 (1)
Geranyl acetone^a^	177 (100), 109 (31), 113 (30), 121 (26), 69 (21), 81 (21), 195 (19), 137 (15), 139 (15), 99 (10), 107 (9), 85 (8), 127 (7), 83 (4)
Benzaldehyde	107 (100), 79 (11)
Benzylalcohol	91 (100), 79 (25)
Limonene^b^	81 (100), 137 (27), 95 (10), 93 (1), 107 (1), 121 (1)

Results for compounds known from literature and from the GC-MS analyses to be emitted by bed bugs. Numbers in brackets show the relative abundance of the different fragments with an E/N-value of ∼135 Td. Fractions due to carbon-13 isotopes have been excluded.

a A mixture of geranyl acetone and neryl acetone (60∶40),

b From [21].

### Experimental Set-up

The feeding chamber was a 2.5 cm length of acrylic tubing (2.6 cm inner diameter) closed at one end by an acrylic plate. The open end was pressed against the arm of a human volunteer, creating a chamber of approximately 13 ml where the bed bugs could feed while their behavioural responses were recorded by a video camera (Sony HDR-SR7E) mounted vertically above the feeding chamber and the arm.

The feeding chamber had two small holes in which teflon tubing (3.2 mm outer diameter) was inserted to ventilate the experimental chamber. The inlet was connected to a charcoal filter and the outlet directly to the PTR-MS vacuum inlet system. In eight experiments, volatile compounds were also collected on adsorption tubes for subsequent analysis on the thermal desorption (TD) GC-MS (see TD GC-MS below). To do so, the outlet from the experimental chamber was split into two: one going to the PTR-MS and the other going through the adsorption tube connected to a vacuum pump (RIPO-pump, Ripo Engineering, Vanlose, Denmark). The flow rate to the PTR-MS was set to be ca. 75 ml/min and the flow to the adsorption tubes was ca. 100 ml/min.

**Table 2:** Examples of individual TD GC-MS and PTR-MS recordings.

		TD GC-MS		PTR-MS	
Fig.	Bed bugs	(*E*)-2-Hexenal	(*E*)-2-Octenal	(*E*)-2-Hexenal	(*E*)-2-Octenal
2A	8♂	290	1,700	510	6,100
2B	4♀4♂	–	–	8,100	2,400
3A	4♀	1.2	n.d.	0	0
3B	4♀4♂	n.d.	3.3	2	8
3C	6♀8♂	440	490	320	160

n.d.: Not found or below detection limit.

Estimated amount of (*E*)-2-hexenal and (*E*)-2-octenal (in ng) emitted in the individual experiments illustrated in Fig. 2 and 3. No TD GC-MS recording was available for the experiment shown in Fig. 2B.

### Experimental Procedure

The bed bugs were transferred in groups of males, females or mixed sex, to the feeding chamber with a piece (1.5×1 cm) of heat treated (200°C for 2 h) glass fibre filter paper (Advantec, Toyo Roshi Kaisha Ltd., Tokyo, Japan). After several tests with two or four animals in a group, it became clear that if the groups were this small, there was little activity. Thus, the preferred group size was eight animals, which was either four of each sex (18 tests) or eight males (eight tests). In five tests, the bed bugs were first kept without access to the arm in the feeding chamber sealed by a piece of plastic (PET, Dupont, Wilmington, DE, USA) tightly fitted to the opening by a short piece of rubber tubing. After 10–15 min the feeding chamber was opened and placed against the arm to allow feeding. In all other tests, the feeding chamber was kept against the arm during the entire experimental period, lasting 10–25 min depending on the amount of activity; if the bed bugs were not active, the recording was interrupted.

### Video Analysis

During play-back of the video recordings the timing of mating attempts were observed. The termination of a mating attempt was not always easy to determine precisely as males sometime left the female slowly, but it was defined as the moment the male gave up pushing the tip of the abdomen underneath the female. It was also noted whether the mating attempt was directed against a female (♂-♀) or a male (♂-♂) or in some cases an already established male-female couple (♂-♀+♂). After feeding, the bed bugs had a tendency to walk on top of the paper facilitating observation, however, in some cases they moved underneath the paper preventing observation of their behaviour. This means that there could have been behavioural reactions that were not observed.

### Chemicals

All chemicals were purchased from commercial suppliers: pentane (>99.5%, VWR International Ltd., Poole, UK), propanal (98%, Sigma, Steinheim, Germany), acetone (>99.9%, Sigma), hexanal (98%, Sigma), heptanal (>97%, Merck, Hohenbrunn, Germany), octanal (99%, Sigma), nonanal (>98%, Merck), decanal (>98%, Sigma), undecanal (>95%, Merck), (*E*)-2-hexenal (>97%, Fluka Chemie, Buchs, Switzerland), (*E*)-2-octenal (94%, Sigma), sulcatone (99%, Sigma), benzaldehyde (>99.5%, Sigma), and benzyl alcohol (>99%, Sigma). Geranyl acetone was purchased from Alfa Aesar (Karlsruhe, Germany), but was only available in a mixture with neryl acetone (60∶40, 97% purity), which means that the relative abundances as indicated in Table 1 contain possible fractions from both compounds. Six additional compounds which have been reported from bed bugs were not available: limonene, (2*E*, 4*E*)-octadienal, (2*E*, 4Z)-octadienal, benzyl acetate [8], acetaldehyde and 2-butanone [4]. Only in the case of limonene, fragmentation pattern was available from the literature [20] and could be included in the analysis.

### PTR-MS

#### Standards and fragmentation pattern

A high sensitivity PTR-MS (Ionicon Analytik, Innsbruck, Austria) was applied in this study. The PTR-MS was operated under standard ion drift tube conditions. The temperature of the drift tube was controlled at 60°C and the pressure was maintained in the range of 2.1–2.2 mbar. A total voltage of 600 V was utilized in the drift tube and the ratio E/N (Electric field per number density of the drift tube buffer gas molecules) was ca. 135 Townsend for all measurements [21]. Since it was difficult to quantify compounds using PTR-MS under a complex matrix especially when compounds with the same molecular weight are present, it was necessary to know the exact fragmentation pattern for all compounds included in the analysis.

All standard compounds were diluted to 0.5 ml/l and 40 ml/l in pentane and injected into clean polyvinyl fluoride (Tedlar) bags, (CEL Scientific Corp., Santa Fe Springs, CA, USA), pre-filled with three litres of clean air using a charcoal filter (Supelpure HC filter, Supelco, Bellefonte, PE, USA), to reach final concentrations of 0.5 ppm and 4 ppm (by volume) of the standards in the Tedlar bags. Pentane is not detectable by the PTR-MS as the proton affinity of pentane is lower than that of water, but despite the high purity (>99.5%) of the pentane, impurities could be present in the same order of magnitude as the standard compounds (0.05–4%). As the amount of pentane in the bags varied it was necessary first to construct a pentane standard curve, based on measurements over a range of concentrations from 320–2900 ppm of pentane. The estimated contribution from pentane together with background measurements from the prefilled Tedlar bags before injecting standards, were subtracted from the measurements with the standards. In this way, it was possible to determine the ion counts for each fraction of a standard compound (at a known concentration of the compound) and to calculate the relative abundance of the fractions for all compounds.

#### Measurements

In an initial experiment, the PTR-MS was running at scan mode of masses between 21 and 200. With a dwell time of 500 ms, each mass was measured every 1.5 min. Based on the results of this experiment and the fragmentation patterns of the standards, a series of masses were chosen for the PTR-MS measurements. It was important to limit the number of masses to measure as a certain dwell time (such as 100 ms) was needed for each mass in order to get stable measurement by PTR-MS. With 40 masses and a dwell time of 100 ms each mass were measured with four seconds interval, ascertaining that even rapidly changing concentrations could be detected. The 100 ms dwell time also meant that the detection limit was around 1 ppb, which was acceptable for the purpose.

#### Analysis

The PTR-MS recordings were analysed by estimating the concentration of (*E*)-2-octenal and (*E*)-2-hexenal based on ion counts for each of the masses 81 and 99 for (*E*)-2-hexenal and masses 67, 109, and 127 for (*E*)-2-octenal, i.e. two estimates for (*E*)-2-octenal and three for (*E*)-2-hexenal. For each of the two compounds, the average concentration was calculated, as basis for an estimate of the expected ion count for the common mass 57. Running a t-test [22] on the ratio between the log-transformed estimated and observed ion count for mass 57 for 20 recordings after each peak (excluding peaks with initial mass 57 ion counts higher than 500,000 where the PTR-MS measurements are no longer linear) gave an indication of whether (*E*)-2-hexenal and (*E*)-2-octenal could account for all of the activity measured for the masses 57, 67, 81, 99, 109, and 127.

For the most prominent peaks of (*E*)-2-hexenal and (*E*)-2-octenal it was possible to estimate the amount of material that had been emitted. After an initial peak, the concentration change over time fitted approximately an exponential function. The fitted function was integrated to the time it reached the average value recorded just before the peak (CurveExpert 1.3, Hyams Development). The sum of recordings in the initial peak was added and a total was calculated.

The amount and ratio of (*E*)-2-hexenal and (*E*)-2-octenal in individual emissions was tested for significant differences between the male-female, male-male, and male-female-male copulation attempts (General Linear Model [22], after log transformation of data).

### TD GC-MS

The major components of the bed bug volatiles collected on adsorption tubes (stainless steel, 8.9 cm long and 0.64 cm outer diameter, packed with Tenax TA (35/60 mesh, Markes International Ltd., Llantrisant, UK) and Carbograph 5D (40/60 mesh, Markes.

**Figure 1:**
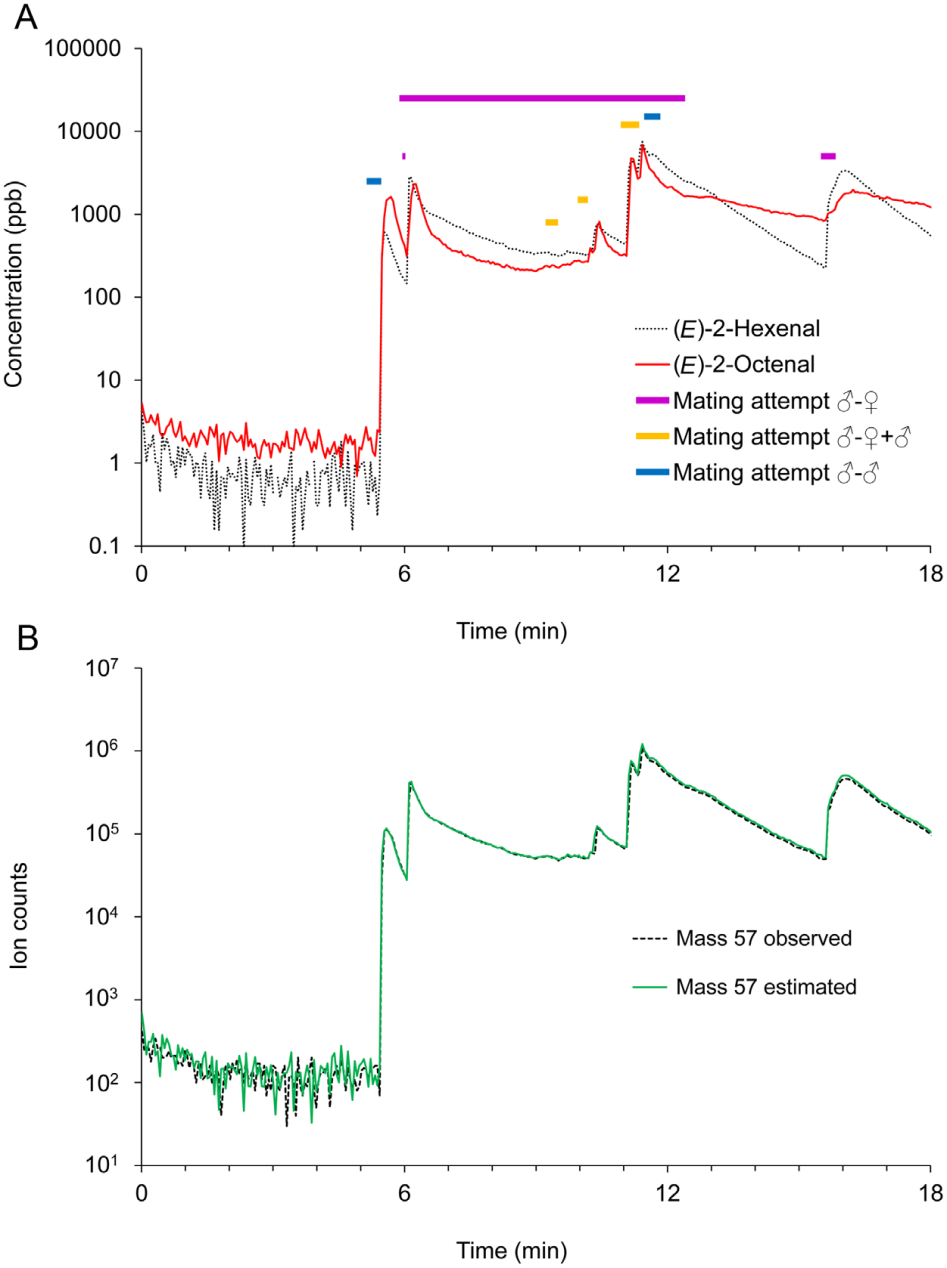
Example of aldehyde emissions and correlation with behavioural observation. Results from a PTR-MS recording on four male and four female bed bugs. The thick horizontal lines indicate mating attempts of male on female (red), male on male (blue) or a second male trying to copulate an already established male-female couple (orange). A. Estimated concentrations of (*E*)-2-hexenal and (*E*)-2-octenal over the entire recording period. B. Observed (dashed black line) and calculated (green line) ion counts for the mass 57 which is produced from both (*E*)-2-hexenal and (*E*)-2-octenal.

International Ltd., UK) in a 3∶5 ratio (Tenax:Carbograph), were analyzed by a coupled gas chromatograph and mass spectrometer (GC 6890N/MSD 5973, Agilent Technologies A/S, Horsholm, Denmark). Before analysis by GC-MS, desorption of components was performed by a 2-step thermal desorber (Turbomatrix ATD, Perkin Elmer, Waltham, MA, USA). First, helium (He) was used to purge the tube for 2 min followed by a desorption for 10 min at 290°C and the desorbed compounds were collected on a cold trap with Tenax TA at −20°C. In the second step, the cold trap was heated to 300°C with a speed of 40°C/sec and the desorbed compounds were transferred to the GC column by a transfer line with a temperature of 250°C (gas He). The temperature of the GC was set to be 50°C and held for 5 min, before it was heated up to 250°C with a speed of 10°C/min and held for 5 min. The column was HP INNOWax (30 m, 0.25 mm ID, 0.25 µm film, Agilent Technologies A/S, Horsholm, Denmark). Concentrations of components were estimated by one-point calibration based on a liquid calibration standard containing the 14 volatiles of interest.

**Figure 2:**
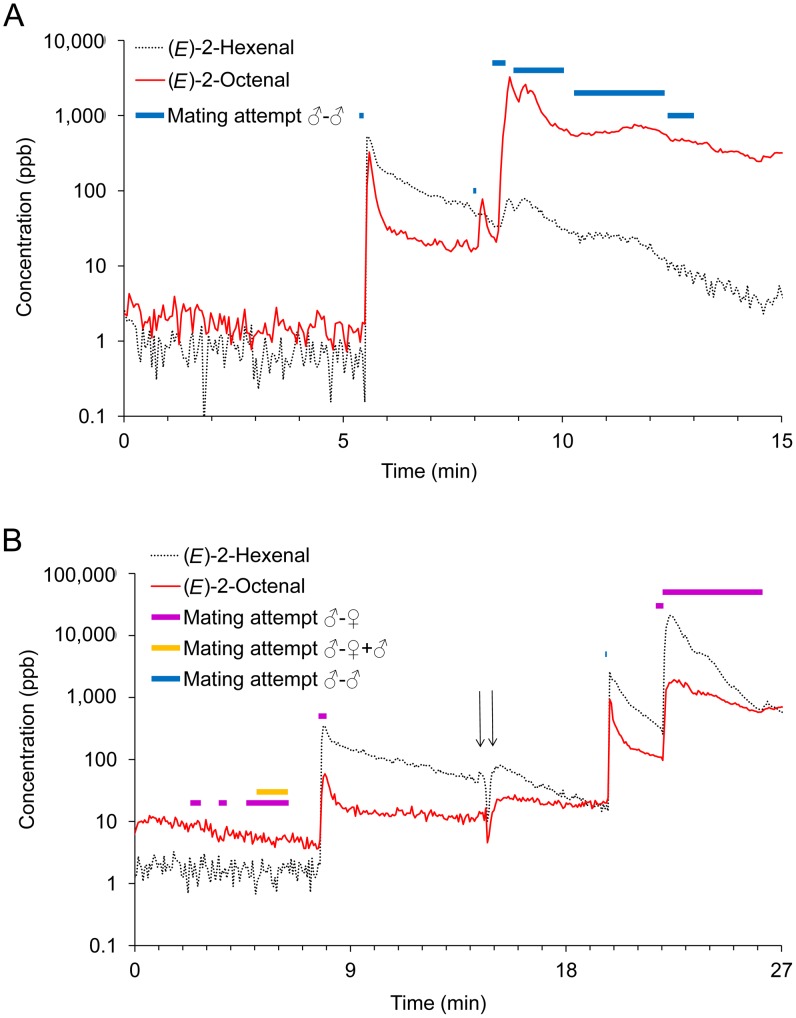
Examples of aldehyde emissions and correlation with behavioural observations. Results from two PTR-MS recordings on either eight males (A) or four males and four females (B). The thick horizontal lines indicate mating attempts of male on female (red), male on male (blue) or a second male trying to copulate an already established male-female couple (orange). The amount of (*E*)-2-hexenal and (*E*)-2-octenal emitted over the entire experimental period of A is also shown in Table 2. In B, the bed bugs were kept in the closed experimental container without access to blood during the first 14 minutes (until the first arrow), then the container was placed on the arm of a human volunteer and the bed bugs were allowed to feed for the last 12 minutes (from the second arrow).

**Figure 3:**
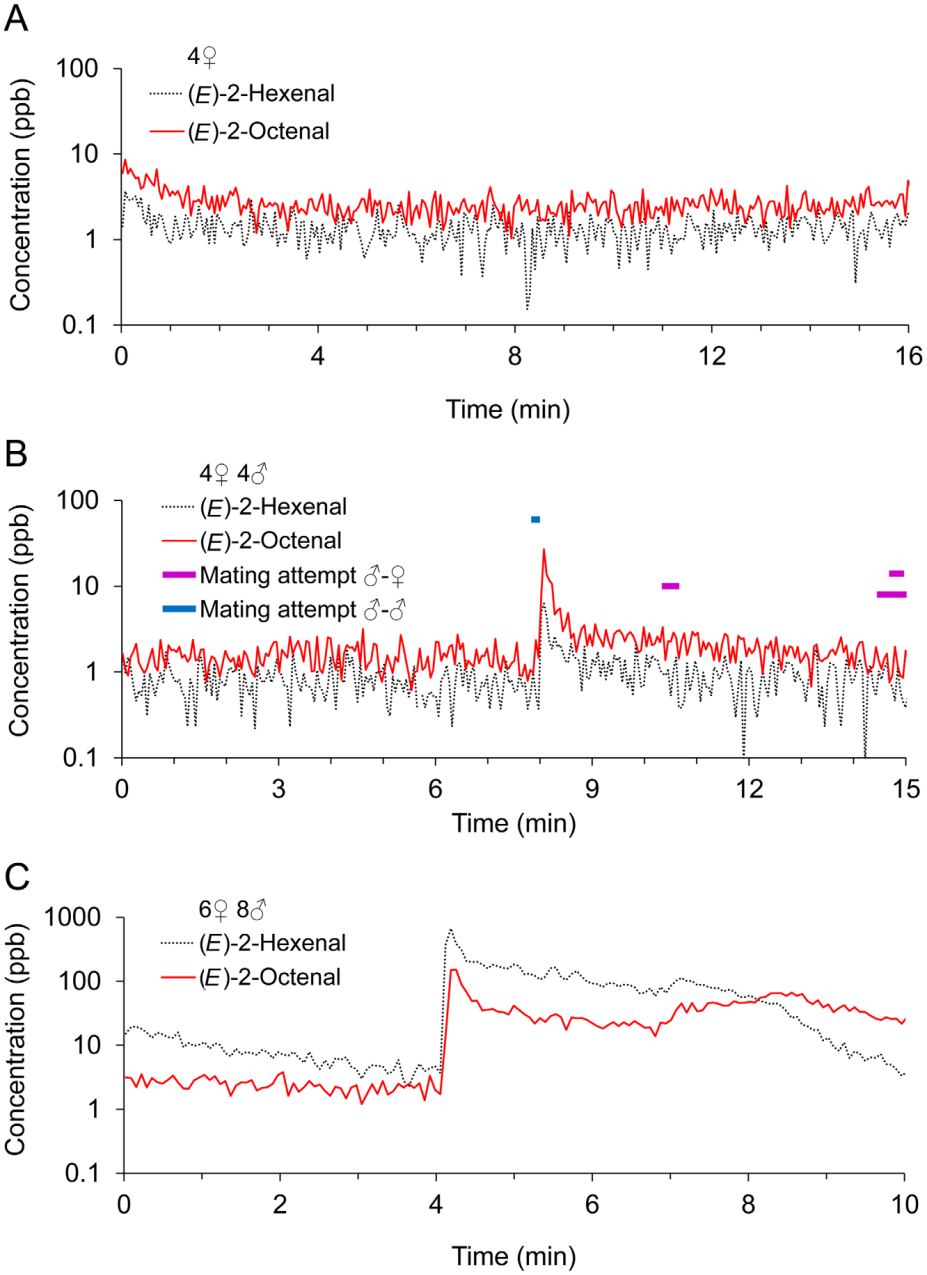
Concentrations of (*E*)-2-hexenal and (*E*)-2-octenal in the PTR-MS recordings from three experiments shown in **Table 2**. In A with four females no mating attempts were observed, in B with four males and four females there was a small peak, and in C there were too many bed bugs for the video analysis. Note that the recording and collection periods were slightly different.

**Figure 4:**
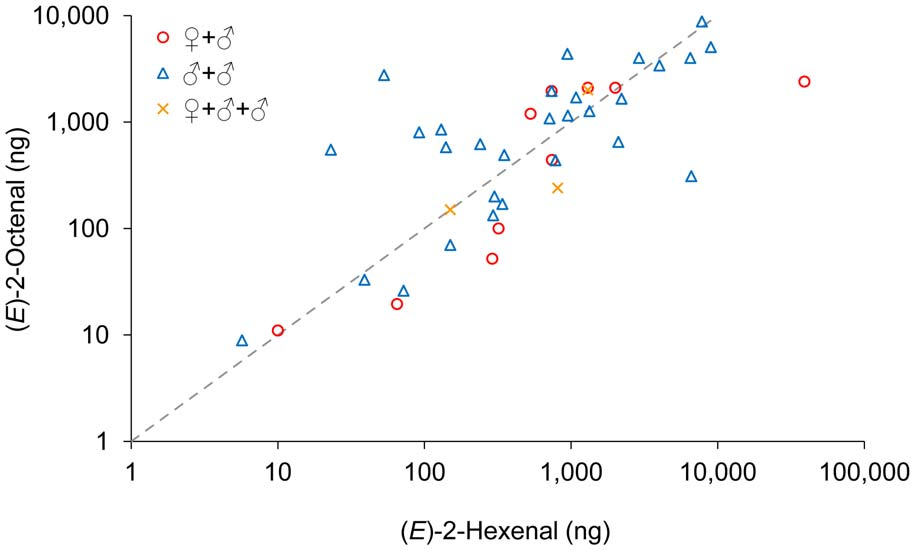
Doses of (*E*)-2-hexenal and (*E*)-2-octenal in individual emissions. All observations are based on PTR-MS recordings combined with video recordings. The dashed line indicates equal amounts of (*E*)-2-hexenal and (*E*)-2-octenal. The ratio of (*E*)-2-hexenal and (*E*)-2-octenal released during the three types of copulation formations were not significantly different from one (♂-♀: *t*_9_ = 1.30; *P* = 0.23; ♂-♂: *t*_28_ = −1.09; *P* = 0.28; ♂-♀-♂: *t*_2_ = 0.53; *P* = 0.65).

## Results

Fragmentation pattern and the relative abundance of fragments measured by the PTR-MS were determined (Table 1) for a range of known and possible bed bug semiochemicals and provided the basis for identification and quantification of compounds released during the bed bug experiments.

In the initial PTR-MS recording (including all ion masses between 21–200) there were several clear peaks for the ion masses that can be attributed to (*E*)-2-hexenal and (*E*)-2-octenal, particularly the ion masses 57, 67, 81, 99, 109, and 127 (Table S1). Only two ion masses (41 and 55) could not be explained directly by these two aldehydes and thus indicated the presence of other minor compounds in the emissions. However, due to the complex fractionation pattern and the presence of (*E*)-2-hexenal or (*E*)-2-octenal in high concentrations, it was not possible to verify the identity of any other compounds.

In all experiments, the ion mass 57, which is produced from both (*E*)-2-hexenal and (*E*)-2-octenal, was always observed in the highest abundance during peaks., A comparison of the observed abundance of ion mass 57 and the abundance expected assuming (*E*)-2-hexenal and (*E*)-2-octenal were the only components in the peaks, showed no significant difference for the 18 peaks analysed (e.g. Fig. 1B), thus suggesting that none of the other compounds producing these ion masses were present in the peaks in significant amount. The only discrepancies between the estimated and the observed values for ion mass 57 were sometimes seen at the first recording cycle of a peak, but this is to be expected as some ion masses in a recording cycle might be measured before the emission whereas other ion masses were measured after the emission.

The most distinct peaks in the PTR-MS recordings were always observed during mating attempts, particularly correlated with the termination of mating attempts (Fig. 1A, 2A and B, and video S1). However, not all mating attempts resulted in (*E*)-2-hexenal and (*E*)-2-octenal peaks, e.g. in Figure 1A there was one long male-female mating attempt lasting more than 6 min that apparently was carried through without the emission of volatiles. Isolated groups of females never emitted any (*E*)-2-hexenal and (*E*)-2-octenal (five tests with four females each, e.g. Fig. 3A), but obviously, they never attempted to mate. Grouping males together resulted in many ♂-♂ copulation attempts and numerous emissions of (*E*)-2-hexenal and (*E*)-2-octenal (36 peaks total in eight tests with eight males in each test, e.g. Fig. 2A). In groups of mixed males and females fewer emissions were observed (23 peaks total in 18 tests with four males and four females in each test, e.g. Fig. 1A and 2B) and only some of these (12 peaks) could be attributed directly to ♂-♀ mating attempts. The remaining emissions could be attributed to either ♂-♂ (eight peaks) or ♂-♀+♂ (three peaks, e.g. Fig. 1A) copulation attempts. Two peaks emitted by unfed bed bugs were observed in the five tests, with an initial test-period without access to the arm (e.g. Fig. 1A). However, this was not enough for statistical analysis.

TD GC-MS analysis of the volatiles collected from groups of males and females also showed that (*E*)-2-hexenal and (*E*)-2-octenal were the most abundant compounds in tests with peaks in the PTR-MS recording (Fig. S1). There were clear differences in the amount of (*E*)-2-hexenal and (*E*)-2-octenal, depending on whether or not peaks were present in the PTR-MS recordings. In Table 2 we have shown comparative results from TD GC-MS and PTR-MS recordings summed up over the entire experimental period, from the experiments also shown in Figure 2 and 3. The amount of (*E*)-2-hexenal and (*E*)-2-octenal measured in the TD GC-MS was of approximately the same order of magnitude as the estimates based on the PTR-MS recordings. Statistical analysis of these comparisons were not possible, as only eight TD GC-MS recordings were obtained with a high variation in the amount of emitted material in individual experiments.

All emission peaks contained both (*E*)-2-hexenal and (*E*)-2-octenal but the amount of material varied over 1000 fold for the two components (Fig. 4). The average amount emitted during ♂-♀copulation attempts was 4,500 ng (*E*)-2-hexenal and 1,000 ng (*E*)-2-octenal, with high variations (SD: 12100 ng (*E*)-2-hexenal; 1000 ng (*E*)-2-octenal). The highest amount observed in a single peak from a ♂-♀ couple was 39,000 ng (*E*)-2-hexenal and 2,400 ng (*E*)-2-octenal (Fig. 4). For ♂-♂ copulation attempts the average emission contained 1,700 ng (*E*)-2-hexenal (SD: 2,600 ng) and 1,600 ng (*E*)-2-octenal (SD: 2,000 ng). Finally, the three emissions during ♂-♀-♂ copulation attempts were on average 750 ng (*E*)-2-hexenal (SD: 580 ng) and 800 ng (*E*)-2-octenal (SD: 1,040 ng). There were no significant differences in the amount of (*E*)-2-hexenal (GLM; *F*_2,39_ = 0.01; *P* = 0.99) or (*E*)-2-octenal (*F*_2,39_ = 0.61; *P* = 0.55) from the different copulation formations.

Also the ratio of the two components varied considerably, but for most peaks the ratio was between 1∶3 and 3∶1, but emissions ranging from 95% (*E*)-2-hexenal to 98% (*E*)-2-octenal were observed (both extremes from ♂-♂ interactions). However, the ratio of the two components was not significantly different between the three copulation formations (*F*_2,39_ = 1.38; *P* = 0.26) and the ratio did not differed significantly from one for any of the three groups (♂-♀: *t*_9_ = 1.30; *P* = 0.23; ♂-♂: *t*_28_ = −1.09; *P* = 0.28; ♂-♀-♂: *t*_2_ = 0.53; *P* = 0.65) as indicated by the dashed line in Figure 4.

## Discussion

Here we have demonstrated the major advantages of PTR-MS analysis combined with video analysis and TD GC-MS for descriptive studies on the use of semiochemicals for intra-specific communication in bed bugs. The combination of methods makes it possible to study in details the temporal pattern of volatiles emitted during specific behavioural activities. The most distinct peaks in the PTR-MS recordings were always observed together with copulation attempts. With the present methods we cannot be absolutely sure which bed bug, e.g. the mounted or the mounting bed bug emitted the pheromones. Since the peaks always occurred close to the termination of a mounting they are considered to have a defensive function to prevent mating attempts, and thus to be emitted by the female or male that was mounted. Previous studies have indicated that male alarm pheromones reduce the risk of homosexual copulation attempts [9], whereas volatiles (so far unknown composition) emitted during heterosexual mating attempts have no effect on the copulation duration [11]. The present results contradict the latter of these findings by showing that (*E*)-2-hexenal and (*E*)-2-octenal emissions from females often lead to termination of heterosexual mating attempts. In the case of ♂-♀+♂ mating attempts we believe the male mounting first emitted the pheromones as it was always the male mounting last that gave up, whereas the initial male continued the mating activities.

In all PTR-MS recordings, the main components of these defensive emissions were (*E*)-2-hexenal and (*E*)-2-octenal, which was also confirmed by the TD GC-MS recordings. Any other undetected component can only have been present at much lower concentration. In contrast to earlier studies [7], [13] there was no significant difference in the ratio or in the amount of (*E*)-2-hexenal and (*E*)-2-octenal regardless of whether they were assigned to a female or a male. Thus, this is not a specific male recognition signal as suggested earlier [9], instead it can protect both males and females against unwanted mountings. The high variation in the ratio of the two components (mostly between 1∶3 and 3∶1, Fig. 4) indicates that the precise composition of the pheromone is not crucial for the biological function. Further, it appears that the bed bugs can regulate the emission rate of the defensive compounds, possibly to balance efficacy with resource conservation. The highest amount emitted at any one time was 40 µg, which is a significant amount for an adult bed bug weighing only around 5 mg before feeding [2], and must be costly to produce.

In a previous study, (*E*)-2-hexenal and (*E*)-2-octenal were identified as essential components of the bed bug aggregation pheromone [12]. Now it is clear that these volatiles have dual functions as defensive chemicals and as part of an aggregation pheromone. Other true bugs (Heteroptera) also emit (*E*)-2-hexenal and (*E*)-2-octenal [23] that function as both alarm and arrestment pheromone depending on the concentration [24], [25]. A third possible function of (*E*)-2-hexenal and (*E*)-2-octenal, relates to the antimicrobial activity of the two compounds that is known from plant science (e.g. [26]). Bed bugs are known to have problems with infections relating to traumatic insemination [27]. Future studies to determine the antimicrobial properties of the bed bug defensive emissions would be interesting as such phenomenon has been observed in other insect genera [28].

The high variation in the composition and the emission rate of the defensive compounds makes it difficult to study bed bug semiochemicals by collecting volatiles over longer periods. The amount of material collected will depend strongly on the mating activity taking place. This explains earlier reports that groups of males are emitting more (*E*)-2-octenal than groups of females [13]. When there are no males present there will be no mating attempts and thus no defensive emissions. In the present study, emission peaks of (*E*)-2-hexenal and (*E*)-2-octenal were never observed from isolated groups of females. However, it is possible that these compounds also function as general alarm pheromones [5]. We occasionally observed elevated concentrations of (*E*)-2-hexenal and (*E*)-2-octenal at the beginning of a recording, just after handling and transferring the bed bugs to the feeding chamber (unpublished results).

Grouping male bed bugs together may lead to homosexual copulation attempts and to the emission of defensive pheromones [9]. In the present study, bed bugs were held in closed confinements to provoke activity. However, it is still not known how common homosexual activity will be in a more natural situation. Likewise, the behavioural response of female bed bugs to heterosexual mating attempts could be more complicated than indicated so far in highly controlled laboratory experiments. Besides defensive emissions and physical avoidance behaviour [11] there are reports that females have lesser tendency to aggregate than males and juveniles [29] possibly to avoid contact with males and thereby prevent copulation attempts. However, female bed bugs need regular blood meals and matings, at least once every month, in order to continue egg production [2]. We did observe copulations that were carried through without the emission of any defensive compounds. Thus, it seems likely there could be situations where the female will accept a mating attempt, e.g. when she actually needs a mating to continue egg production.

The method of combining PTR-MS and video analysis has demonstrated its high potential for studies of chemical ecology, particularly in cases where the semiochemicals are known, so that only few ions need to be measured, increasing both sensitivity and temporal resolution. The system works best with compounds that are highly volatile as concentration changes are most easily observed. Bed bugs represent a fine model system for such analysis because they emit highly volatile semiochemicals in relatively high amounts (some are detectable by the human nose). With the set-up applied here (dwell time 100 ms) the detection limit of the PTR-MS reached below 1 ppb, but this was not sensitive enough to detect all compounds. It might be possible that more detailed analysis, focussing on fewer ions could give a more complete picture of the bed bug chemical ecology. One disadvantage of the PTR-MS system is that the final identification of the volatile compounds has to rely on other types of analysis, such as TD GC-MS, but it is possible to combine the two methods in a set-up as described here.

## Supporting Information

Figure S1 **Gas chromatogram from a group of bed bugs.** Representative TD GC-MS trace of head space sample from eight males (from the experiment also shown in Fig. 2A).
(PDF)

Table S1 **PTR-MS recordings from five individual peaks in experiments where all masses in the range 21–200 were recorded.** For each peak those masses have been included where three subsequent recordings during the peak were all higher than the average, plus two times the standard deviation, of the 15 previous recordings. The average of those three peak recordings was calculated and listed according to their abundance relative to the most abundant mass (mw 57). The column to the right indicates the possible compounds that each mass could be attributed to and their relative abundance for each compound. (*E*)-2-Hexenal and (*E*)-2-octenal have been highlighted as they can explain most of the observed masses. Masses that can be attributed to carbon-13 isotopes have been written in italics. In these experiments the recording cycle was 1.5 minutes meaning that the relative abundance of the ions was difficult to interpret.
(DOC)

Video S1 **Sequence combining video recording of copulation attempts and the estimated concentrations of (** ***E*** **)-2-hexenal and (** ***E*** **)-2-octenal measured by PTR-MS.** A male bed bug first attempts to copulate a female bed bug. The copulation attempt is given up without emission of chemicals as another male approach the couple. The first male rapidly mounts the second male. Red arrow indicates estimated time of emission of defensive chemicals where after the copulation attempts is given up. The concentrations of (*E*)-2-hexenal and (*E*)-2-octenal have been estimated by interpolating between measurements (5.5 seconds interval).
(MP4)
