# Supplementary figures and images for: Real-Time Measurement of Volatile Chemicals Released by Bed Bugs during Mating Activities
Source: PLoS One. 2012 Dec 5;7(12):e50981. doi: 10.1371/journal.pone.0050981 (PMC3515544; doi:10.1371/journal.pone.0050981)

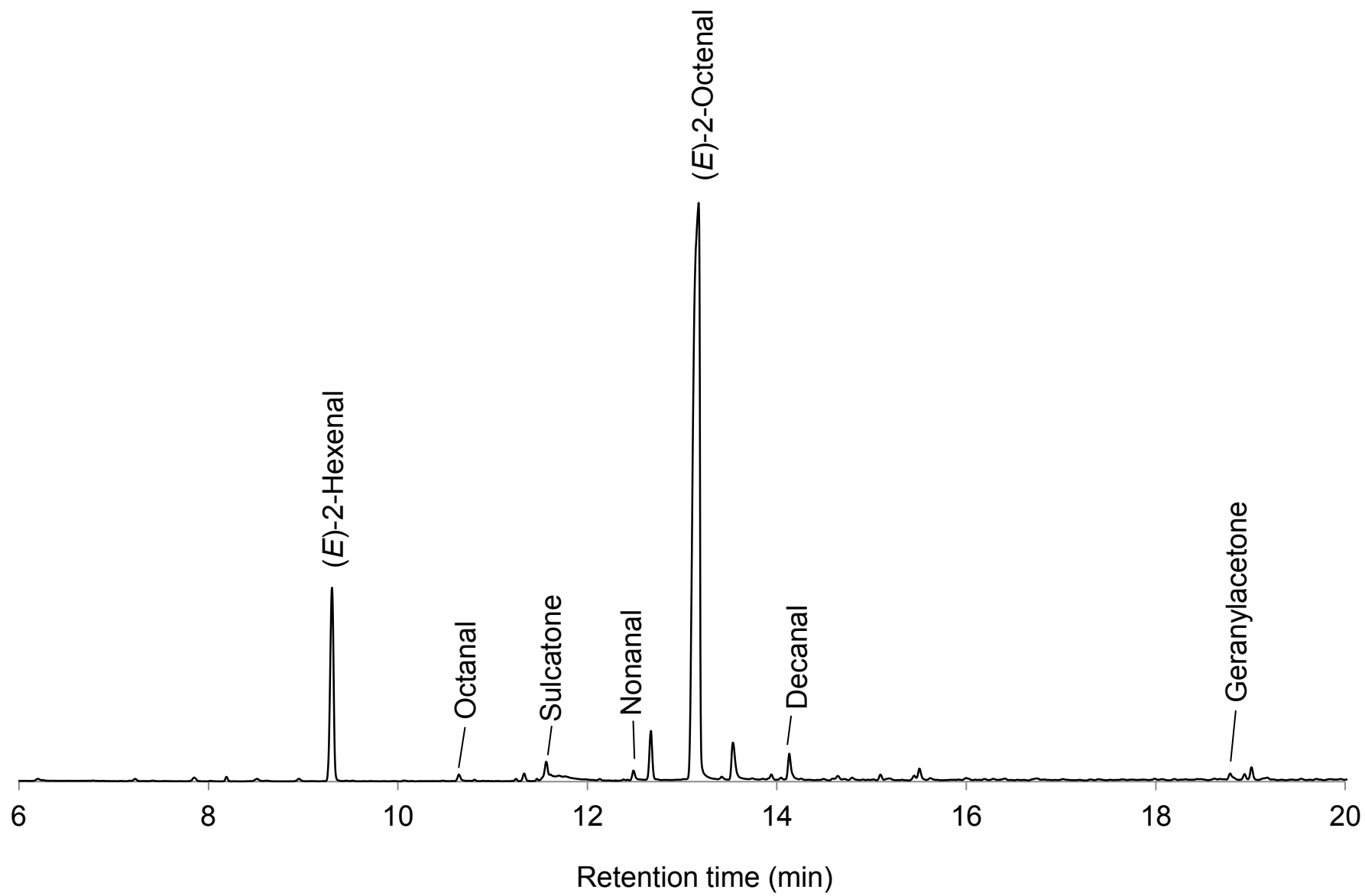

Supplement: Figure S1 — Gas chromatogram from a group of bed bugs. Representative TD GC-MS trace of head space sample from eight males (from the experiment also shown in Fig. 2A). (PDF) [file pone.0050981.s001.pdf]
