# Supplementary material for: Real-Time Measurement of Volatile Chemicals Released by Bed Bugs during Mating Activities
Source: PLoS One. 2012 Dec 5;7(12):e50981. doi: 10.1371/journal.pone.0050981 (PMC3515544; doi:10.1371/journal.pone.0050981)
Supplement: Table S1 — PTR-MS recordings from five individual peaks in experiments where all masses in the range 21–200 were recorded. For each peak those masses have been included where three subsequent recordings during the peak were all higher than the average, plus two times the standard deviation, of the 15 previous recordings. The average of those three peak recordings was calculated and listed according to their abundance relative to the most abundant mass (mw 57). The column to the right indicates the possible compounds that each mass could be attributed to and their relative abundance for each compound. (E)-2-Hexenal and (E)-2-octenal have been highlighted as they can explain most of the observed masses. Masses that can be attributed to carbon-13 isotopes have been written in italics. In these experiments the recording cycle was 1.5 minutes meaning that the relative abundance of the ions was difficult to interpret. (DOC) [file pone.0050981.s002.doc]

**Table S 1. PTR-MS recordings from 5** individual peaks in experiments where all masses between 21-200 were recorded.

| **Ion mass** | **Relative abundance** | | | | | **Possible compounds** |
| --- | --- | --- | --- | --- | --- | --- |
| **57** | 100.0 | 100.0 | 100.0 | 100.0 | 100.0 | **(*E*)-2-Hexenal (100); (*E*)-2-Octenal (53);** Nonanal (24) |
| **99** | 17.4 | 15.5 | 54.6 | 24.0 | 16.8 | **(*E*)-2-Hexenal (23);** Geranyl acetone (10) |
| **109** | 16.5 | 49.1 | 30.2 | 10.1 | 5.7 | **(*E*)-2-Octenal (100);** Sulcatone (100); Geranyl acetone (31) |
| **81** | 14.4 | 13.0 | 24.9 | 14.9 | 14.6 | **(*E*)-2-Hexenal (21);** Geranyl acetone (21); Decanal (8); Undecanal (3) |
| **127** | 5.4 | 31.1 | 18.3 | 4.4 | 1.9 | Sulcatone (25); **(*E*)-2-Octenal (33);** Geranyl acetone (7) |
| **43** | 4.9 | 4.6 | 4.4 | 3.7 | 4.2 | Undecanal (66); **(*E*)-2-Hexenal (6)** |
| **67** | 3.8 | 7.2 | 6.9 | 2.3 | 1.5 | **(*E*)-2-Octenal (28);** Nonanal (4); Octanal (2); Sulcatone (1) |
| **58** | 3.6 | 3.3 | 3.3 | 3.5 | 3.4 | *57+1* |
| **110** | 1.5 | 7.2 | 2.6 | 0.7 | 0.5 | *109+1* |
| **55** | 1.4 | 1.8 | 2.3 |  |  | Heptanal (100); Hexanal (100); Decanal (92); Undecanal (100); Nonanal (32); Octanal (11) |
| **100** | 1.2 | 1.0 | 3.8 | 1.6 | 1.2 | *99+1* |
| **82** | 1.0 |  | 1.7 | 1.1 | 1.0 | *81+1* |
| **41** | 0.8 | 1.1 | 0.6 | 0.5 |  | Octanal (47) |
| **59** | 0.7 | 4.8 | 1.4 |  | 0.5 | Acetone (100); Propanal (100); **(*E*)-2-Octenal (4)** |
| **83** | 0.6 | 0.6 | 1.2 | 0.5 |  | Decanal (100); Hexanal (74); Undecanal (38); Nonanal (33); Geranyl acetone (4); **(*E*)-2-Octenal (2)** |
| **128** | 0.5 | 2.7 | 1.6 |  |  | *127+1* |
| **98** |  |  | 0.6 |  |  |  |
| **29** |  | 0.8 |  |  |  |  |
| **75** |  |  | 0.6 |  |  |  |
